# Risk of noise-induced hearing loss in the spine surgeon

**DOI:** 10.1016/j.xnsj.2023.100297

**Published:** 2023-11-24

**Authors:** Matthew H. Meade, Stephanie A. Kwan, Mark E. Michael, Nicholas J. Minissale, Levi Buchan, Jeffrey R. Gleimer, Barrett I. Woods, Christopher Kepler

**Affiliations:** a Division of Orthopaedic Surgery, Jefferson Health – New Jersey, Stratford, NJ, United States; b Regional Orthopedic Professional Association, Cherry Hill, NJ, United States; c The Rothman Institute at Thomas Jefferson University, Department of Orthopaedic Spine Surgery, 925 Chestnut St., Philadelphia, PA 19107, United States

**Keywords:** Noise, Noise-induced hearing loss, Occupational hazard, Spine, Decibel, Hearing loss

## Abstract

**Background:**

Occupation-related noise-induced hearing loss (NIHL) has both negative economic and quality of life implications. The risk spine surgeons undertake in regards to NIHL during operative intervention is unknown. Governing bodies, including the National Institute for Occupational Safety and Health, have recommended exposure limits not to exceed 85 decibels (dB) over 8 hours. The purpose of this study is to characterize noise exposure to spine surgeons in the operating room (OR).

**Methods:**

Prospective collection of intraoperative recordings of spinal surgeries (cervical and thoracic/lumbar) was undertaken. Data gathered included procedure, operative duration, presence of background music, and noise information. Noise information included maximum decibel level (MDL), Peak level (LCPeak), Equivalent continuous sound pressure level, time weighted average (TWA), dose, and projected dose. Noise measurements were compared with baseline controls with and without music (empty ORs).

**Results:**

Two hundred seven noise recordings were analyzed. One hundred eighteen of those being spinal surgeries, 49 baseline recordings without music, and 40 with music. Maximum decibel level reached a maximum value of 111.5 dBA, with an average amongst surgical recordings of 103 dBA. Maximum decibel level exceeded 85 dBA in 100% of cases and was greater than 100 dBA in 78%. The maximum LCPeak recorded was 132.9 dBC with an average of 120 dBC. Furthermore, the average dose was 7.8% with an average projected dose of 26.5%. The highest dose occurred during a laminectomy at 72.9% of daily allowable noise. Maximum projected dose yielded 156% during a 3-level anterior cervical discectomy and fusion.

**Conclusions:**

Spine surgeons are routinely exposed to damaging noise levels (>85 dBA) during operative intervention. With spine surgeons often performing multiple surgeries a day, the cumulative risk of noise exposure cannot be ignored. The synergistic effects of continuous and impact noise places spine surgeons at risk for the development of occupation-related NIHL.

## Introduction

According to the Centers for Disease Control and Prevention, hearing loss is the third most common chronic physical condition among adults in the United States [1]. Approximately 24% of hearing difficulty is caused by occupational exposures [2]. Noise-induced hearing loss (NIHL) caused by workplace noise exposure is a significant cause of health problems worldwide with occupational noise exposure responsible for at least 16% of disabling hearing loss in adults [3], [4], [5], [6]. Noise-induced hearing loss can impact one's communication and can negatively impact quality of life [3,^7^,8]. Additionally, impacts of occupational noise exposure and hearing damage can cause tremendous financial and disease burden with estimated annual compensation for NIHL in the US at $242.2 million dollar's [3,^5^,8]. In response, the National Institute for Occupational Safety and Health (NIOSH) has recommendations which state that the recommended exposure limit for occupational noise is 85 dBA over an 8-hour period [9].

To date, there is sparse literature and minimal understanding of the occupational noise hazards present to spine surgeons in the operating room (OR). Spine surgery itself commonly involves power driven tools and impact devices to decompress neurologic structures or mobilize bony anatomy. These tools are an integral part of spine surgery but have potential to cause iatrogenic injury to the patient, OR staff, and surgeons [10]. Overall, there is limited understanding of the possible significant exposure to occupational sound pollution in the operating suite. Noise exposure in the OR has been poorly reported on and data regarding the risk of NIHL is conflicting [11,12].

The purpose of this study is to address the shortcomings in the literature and describe noise exposure to spine surgeons across the most common procedures. This study will help to further understand the risks spine surgeons encounter with regards to noise pollution in the OR and could allow future measures to be put on place in order to mitigate the risk of NIHL.

## Materials and methods

Following Institutional Review Board exemption, a prospective review was conducted by collecting intraoperative recordings with an external microphone in the OR. The NIOSH iOS (Apple) based sound level meter application to collect noise exposure was used in conjunction with an external, iMM-6 calibrated, microphone (Dayton Audio) [13].

The SLM application was developed as a practical application, in order to collect real-time noise exposure utilizing a smartphone device and has been shown to accurately capture noise within 1 decibel of a reference sound [14,15]. Intraoperatively, the microphone was worn by the attending or resident staff in the front chest pocket to replicate the surgeons hearing zone. When surgical lead was done, the microphone was worn in the chest pocket. The microphone was worn beneath the sterile surgical gown to maintain appropriate surgical sterility throughout the procedure. Additionally, the microphone was secured to the surgical lead/surgical scrubs with silk tape to limit friction from the sterile surgical gown. Recordings were begun before surgical scrub and concluded upon removal of the surgical gown.

In total, 118 recordings were made of in-hospital spine surgery cases. Forty-nine “baseline” levels were obtained using 30-second recordings in the OR before surgery, without OR personnel present or music playing. An additional 40 “baseline with music” levels were obtained in the same fashion. The “baseline with music” recordings had background music playing at a level equivalent to that used during operative intervention. Intraoperative recordings quantified 6 noise characteristics including maximum dB level (MDL), Peak level (LCPeak), equivalent continuous sound pressure level (Laeq), time weighted average (TWA), dose, and projected dose.

Maximum decibel level is the highest sound level encountered using an A-weighted dB scale (dBA). A-weighted scales are the most commonly used and adjust the sound pressure level readings to reflect the sensitivity of the human ear. A-weighted scales effectively evaluate only the frequencies at which the average person can hear thus discriminating against lower frequencies.

Peak level is the maximum absolute value reached instantaneously by the sound pressure using a C-weighted dB scale. Peak level is used for occupational noise measurement where loud bangs are present or there is impulse noise. The C-weighted frequency assess the effect of low frequency sounds on the human ear and captures impulsive noise or the peak sound pressure. The C-weighted sound level measures uniformly over its frequency range. This is important as the response of the human ear varies with sound level and C-weighted scales can assist in capturing noise not as well evaluated by dBA. The major distinction between MDL and LCPeak is the scale used (dBA vs. dBC). Equivalent continuous sound pressure level, or “average sound level,” demonstrates the average noise level for the period of exposure.

Time weighted average is the average dB level projected over an 8-hour time period on an A-weighted scale.

Dose is the percentage of noise exposure for the given recording as a percentage of daily allowable noise exposure. Cumulative noise dose should not exceed 100% in a given 24-hour period as the cumulative dose of acetaminophen should not exceed 4000mg over a 24-hour period.

Projected dose, is the measured noise dose projected forward over 8-hour. All dB levels were measured using an A-weighted scale (dBA) with the exception of LCpeak which used a C-weighted scale (dBC). C-weighted scales are used to register short but clinically relevant sound pressure peaks not identified by dBA [16]. Other characteristics recorded included procedure, procedure duration, and presence of background music.

Surgeries included all spinal regions and approaches. Typical spinal surgery cases evaluated and included anterior cervical discectomy and fusion (ACDF), cervical disc replacement (CDR), and posterior cervical decompression and fusion (PCDF). Cervical procedures included anterior cervical discectomy and fusion (ACDF), cervical disc replacement (CDR), and posterior cervical decompression and fusion (PCDF). Thoracic/Lumbar procedures include thoracic laminectomies with arthrodesis and posterior instrumentation, lumbar discectomies, laminectomies, laminectomies with posterior arthrodesis and instrumentation (PLDF), and lumbar interbody fusions (of any direction) with (PLDF). Surgeries were performed by 5 fellowship trained spine surgeons at 2 hospitals.

Baseline and operative recordings were compared using independent *t*-tests or ANOVA tests depending on normality. The *t*-tests were used to compare continuous data for groups of 2, ANVOA for groups of 3, and Fisher's Exact were used for categorical data. p-values less than .05 were deemed significant and all statistical analyses were done using RStudio (version 4.1.2, Vienna, Austria).

## Results

Two hundred seven noise recordings were analyzed consisting of 118 spine surgeries, 49 baseline recordings without music, and 40 baseline recordings with music (Tables 1 and 2). There were 40 cervical procedures (33.9%) and 78 thoracic/lumbar cases (66.1%). Cervical procedures included 32 ACDFs (80%), 3 CDRs (7.5%), and 5 PCDFs (12.5%). Thoracic/lumbar procedures consisted of 29 lumbar interbody fusions of any direction (37.2%), 20 laminectomies (25.6%), 17 PLDFs (21.8%), 9 microdiscectomies (11.5%), and 3 thoracic laminectomies with arthrodesis and posterior instrumentation (PTDF) (3.9%). Surgical duration averaged 131 minutes (95% CI, 118–145 minutes) with cervical (mean = 129 minutes; 95% CI, 105–153 minutes) procedures shorter than thoracic/lumbar (mean = 132 minutes; 95% CI, 116–149 minutes). One hundred eleven cases (94.1%) had background music whereas the remainder (5.9%) did not.

**Table 1:** Comparison of baseline recordings (without music) to all intraoperative recordings

	Baseline (without music)		Surgery		p value
	N = 49		N = 118		
	Mean (SD)*	*95% CI*†	Mean (SD)*	*95% CI*†	
Duration (minutes)	0.5 (0.0005)		131 (69.3)	131 [118;145]	
MDL (dBA)	65.2 (12.8)	65.2 [61.5;68.8]	103 (4.4)	103 [102;104]	<.001
LCPeak (dBC)	85.8 (15.3)	85.8 [81.4;90.1]	120 (4.8)	120 [120;121]	<.001
Laeq (dBA)	57.2 (5.5)	57.2 [55.6;58.8]	78.6 (3.9)	78.6 [77.9;79.3]	<.001
TWA (dBA)	22 (14.8)	22 [17.7;26.3]	70.2 (5.4)	70.2 [69.2;71.1]	<.001
Dose (%)	0 (0)	0 [0;0]	7.8 (20.5)	7.8 [4.1;11.5]	<.001
Projected dose (%)	10.6 (14.6)	10.6 [6.4;14.8]	26.5 (31.3)	26.5 [20.8;32.2]	<.001

MDL, maximum decibel level; LCPeak, peak level; Laeq, equivalent continuous sound pressure level; TWA, time weighted average. Duration includes surgical recordings which were begun before surgical scrub and concluded upon removal of the surgical gown in minutes. The MDL, LCPeak, Laeq, TWA, dose, and projected dose at baseline and during surgical procedures,

⁎ Values are presented as mean with standard deviation in parentheses.

† 95% confidence intervals are presented in brackets.

**Table 2:** Comparison of baseline recordings with music to all intraoperative recordings

	Baseline with music		Surgery		p value
	N = 40		N = 118		
	Mean (SD)*	*95% CI*†	Mean (SD)*	*95% CI*†	
Duration (minutes)	0.5 (0.0005)		131 (69.3)	131 [118;145]	
MDL (dBA)	66.9 (3.7)	66.9 [65.2;68.6]	103 (4.4)	103 [102;104]	<.001
LCPeak (dBC)	87.2 (8.6)	87.2 [83.3;91.1]	120 (4.8)	120 [120;121]	<.001
Laeq (dBA)	59.3 (3.9)	59.3 [57.5;61.1]	78.6 (3.9)	78.6 [77.9;79.3]	<.001
TWA (dBA)	24.2 (8.6)	24.2 [22.1;26.3]	70.2 (5.4)	70.2 [69.2;71.1]	<.001
Dose (%)	0 (0)	0 [0 ; 0]	7.8 (20.5)	7.8 [4.1;11.5]	<.001
Projected dose (%)	12.2 (6.6)	12.2 [10.1;14.3]	26.5 (31.3)	26.5 [20.8;32.2]	<.001

MDL, maximum decibel level; LCPeak, peak level; Laeq, equivalent continuous sound pressure level; TWA, time weighted average. Duration includes surgical recordings which were begun before surgical scrub and concluded upon removal of the surgical gown in minutes. The MDL, LCPeak, Laeq, TWA, dose, and projected dose at baseline and during surgical procedures.

⁎ Values are presented as mean with standard deviation in parentheses.

† 95% confidence intervals are presented in brackets.

Overall, the greatest MDL was recorded during a 52-minute L5–S1 microdiscectomy at 111.5 dBA. The maximum LCPeak was recorded in an 84-minute L5–S1 laminectomy at 132.9 dBC. The maximum dose occurred during 252-minute L2–4 laminectomy with a value of 72.9% whereas the highest projected dose was 156% during 132-minute C4–7 ACDF.

Noise levels for all procedures were significantly greater compared with control recordings (both without music and with music) for MDL, LCpeak, Laeq, TWA, dose, and projected dose (p < .001; Tables 1 and 2). Maximum decibel level yielded an average of 103 dBA for all recordings (95% CI, 102–104 dBA). Maximum decibel level was greater than 85 dBA for 100% of surgeries and was more than 100 dBA in 92 (78%) of recordings. Peak level yielded an average of 120 dBC [95% CI, 120–121 dBC] whereas Laeq was 78.6 dBA (95% CI, 77.9–79.3 dBA). Mean TWA was 70.2 dBA (95% CI, 69.2–71.1 dBA) with an average dose of 7.8% (95% CI, 4.1%–11.5%) and projected dose of 26.5% (95% CI, 20.8%–32.2%). Notably, 19 cases (16.1%) had a projected dose greater than 50% with 5 of those (4.2%) over 100%.

Subsequently, a subgroup analysis was performed comparing cervical and thoracic/lumbar cases individually to baseline noise levels (Table 3 and Figure). All measures were significantly greater compared with controls for MDL, LCPeak, Laeq, TWA, dose, and projected dose. Maximum decibel level for both cervical and thoracic/lumbar cases averaged 103 dBA (95% CI, 101–104 dBA; 95% CI, 102–105 dBA). The highest recorded MDL for cervical procedures was 111.3 dBA whereas for thoracic/lumbar surgeries it was 111.5 dBA. Similarly, average LCPeak for cervical and thoracic/lumbar interventions was identical at 121 dBC (95% CI, 119–122 dBC; 95% CI, 119–121 dBC). Maximum values for LCPeak were 130.7 dBC in cervical surgeries and 132.9 dBC in thoracic/lumbar. LAeq was, on average, less during cervical surgeries as compared with thoracic/lumbar interventions with corresponding values of 77.9 dBA (95% CI, 76.8–79 dBA) and 78.9 dBA (95% CI, 78–79.8 dBA). Time weighted average for cervical cases generated 69.1 dBA (95% CI, 67.4–70.8 dBA) whereas thoracic/lumbar cases were 70.7 dBA (95% CI, 69.5–71.9 dBA). Dose for cervical and thoracic/lumbar interventions yielded 5.2% (95% CI, 3.1%–7.2%) and 9.2% (95% CI, 3.6%–14.7%) respectively. Lastly, projected dose for the cervical cohort showed a mean of 21.5% (95% CI, 12.1%–30.9%) whereas thoracic/lumbar cases were 29.1% (95% CI, 21.8%–36.4%). Cervical procedures were also compared with thoracic/lumbar interventions with no difference amongst any variable (Table 4).

**Table 3:** Comparison of cervical and thoracolumbar surgeries to baseline recordings

	Cervical		Thoracic/lumbar		Baseline without music	Baseline with music	p value
	N = 40		N = 78		N = 49	N = 40	
	Mean (SD)*	*95% CI*†	Mean (SD)*	*95% CI*†			
Duration (minutes)	129 (65.3)	129 [105;153]	132 (71.5)	132 [116;149]	0.5 (0.0005)	0.5 (0.0005)	.820
MDL (dBA)	103 (4.3)	103 [101;104]	103 (4.4)	103 [102;105]	65.2 (12.8)	66.9 (3.7)	<.001
LCPeak (dBC)	121 (4.5)	121 [119;122]	120 (4.9)	120 [119;121]	85.8 (15.3)	87.2 (8.6)	<.001
Laeq (dBA)	77.9 (3.6)	77.9 [76.8;79]	78.9 (4)	78.9 [78;79.8]	57.2 (5.5)	59.3 (3.9)	<.001
TWA (dBA)	69.1 (5.3)	69.1 [67.4;70.8]	70.7 (5.4)	70.7 [69.5;71.9]	22 (14.8)	24.2 (8.6)	<.001
Dose (%)	5.2 (6.5)	5.2 [3.1;7.2]	9.2 (24.7)	9.2 [3.6;14.7]	0 (0)	0 (0)	.016
Projected Dose (%)	21.5 (29.4)	21.5 [12.1;30.9]	29.1 (32.2)	29.1 [21.8;36.4]	10.6 (14.6)	12.2 (6.6)	.001

MDL, maximum decibel level; LCPeak, peak level; Laeq, equivalent continuous sound pressure level; TWA, time weighted average; ACDF, anterior cervical discectomy and fusion; CDR, cervical disc replacement; PCDF, posterior cervical decompression and fusion. Duration includes surgical recordings which were begun before surgical scrub and concluded upon removal of the surgical gown in minutes. The MDL, LCPeak, Laeq, TWA, dose, and projected dose baseline and during thoracolumbar procedures and cervical procedures individually. Cervical procedures included ACDF, CDR, and PCDF. Thoracic/Lumbar procedures include thoracic laminectomies with arthrodesis and posterior instrumentation, lumbar discectomies, laminectomies, laminectomies with posterior arthrodesis and instrumentation, and lumbar interbody fusions (of any direction) with posterior arthrodesis and instrumentation.

⁎ Values are presented as mean with standard deviation in parentheses.

† 95% confidence intervals are presented in brackets.

**Figure:**
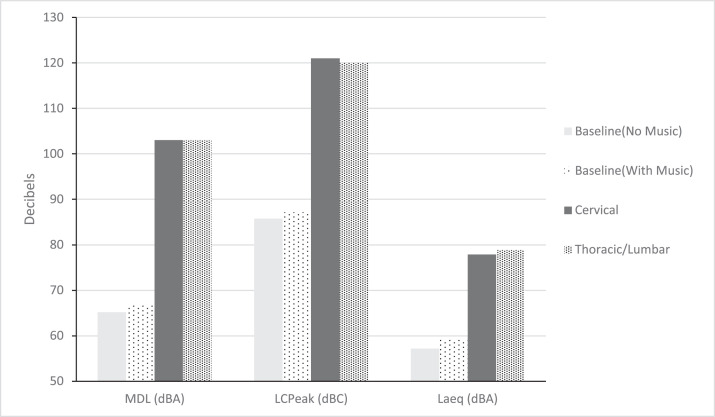
A comparison of baseline recordings without music, baseline recordings with music, cervical recordings and thoracic/lumbar recordings for average MDL, average LCPeak and average Laeq. Decibels on an A-weighted scale are noted dBA. Decibels on a C weighted scale are noted dBC. *Note: Per the National Institute for Occupational Safety and Health (NIOSH) recommended exposure limitations, exposure of 100 dBA should not exceed 15 minutes in a given 24-hour time period. MDL, maximum decibel level; LCPeak, peak level; Laeq, equivalent continuous sound pressure level.

**Table 4:** Comparison of cervical cases to thoracolumbar cases

	Cervical		Thoracic/lumbar		p value
	N=40		N=78		
	Mean (SD)*	*95% CI*†	Mean (SD)*	*95% CI*†	
Duration (minutes)	129 (65.3)	129 [105;153]	132 (71.5)	132 [116;149]	.814
MDL (dBA)	103 (4.3)	103 [101;104]	103 (4.4)	103 [102;105]	.449
LCPeak (dBC)	121 (4.5)	121 [119;122]	120 (4.9)	120 [119;121]	.779
Laeq (dBA)	77.9 (3.6)	77.9 [76.8;79]	78.9 (4)	78.9 [78;79.8]	.168
TWA (dBA)	69.1 (5.3)	69.1 [67.4;70.8]	70.7 (5.4)	70.7 [69.5;71.9]	.121
Dose (%)	5.2 (6.5)	5.2 [3.1;7.2]	9.2 (24.7)	9.2 [3.6;14.7]	.183
Projected dose (%)	21.5 (29.4)	21.5 [12.1;30.9]	29.1 (32.2)	29.1 [21.8;36.4]	.200

MDL, maximum decibel level; LCPeak, peak level; Laeq, equivalent continuous sound pressure level; TWA, time weighted average; ACDF, anterior cervical discectomy and fusion; CDR, cervical disc replacement; PCDF, posterior cervical decompression and fusion. Duration includes surgical recordings which were begun before surgical scrub and concluded upon removal of the surgical gown in minutes.

The MDL, LCPeak, Laeq, TWA, dose, and projected dose during thoracolumbar procedures and cervical procedures individually. Cervical procedures included ACDF, CDR, and PCDF. Thoracic/lumbar procedures include thoracic laminectomies with arthrodesis and posterior instrumentation, lumbar discectomies, laminectomies, laminectomies with posterior arthrodesis and instrumentation, and lumbar interbody fusions (of any direction) with posterior arthrodesis and instrumentation.

⁎ Values are presented as mean with standard deviation in parentheses.

† 95% confidence intervals are presented in brackets.

## Discussion

Occupational hearing loss is one of the most common work-related illnesses in the US with at least 22 million workers exposed to hazardous noise levels [9]. Occupational Safety and Health Administration and NIOSH have specific recommended exposure limits of noise which are deemed hazardous for all occupations (Table 5). The NIOSH currently sets an occupational noise exposure limit of 85 dBA over 8 hours [17]. It is imperative to understand that these guidelines assume that an individual has no noise exposure throughout the remainder of the day. The Centers for Disease Control and Prevention, United States Environmental Protection Agency, and World Health Organization also recognize that prolonged exposure to noise over 70 dBA may start to damage hearing [18]. The vast impact that hearing impairment can have on occupational safety and health cannot be overstated. Zwerling et al. [19] has demonstrated that hearing loss is associated with a substantially increased risk of occupational injury.

**Table 5:** National Institute for Occupational Safety and Health (NIOSH) recommended exposure limitations (RELs) in 24-hour time period

Decibels (dBA)	Permissible exposure in 24 h
85	8 h
88	4 h
91	2 h
94	1 h
97	30 min
100	15 min
103	7 min and 30 s

Previous studies have shown that operating suite noise puts OR staff and patients at risk for NIHL [20,21]. National Institute for Occupational Safety and Health reports that approximately 13% of healthcare workers are exposed to hazardous noise [7,22]. This value may underestimate the risk to those that spend a large portion of their work day in the OR where substantial noise can be generated from surgical instruments [23]. Willet et al. [20] evaluated a variety of orthopedic staff present in the OR and demonstrated that 50% of subjects demonstrated NIHL, with increased years of exposure correlated with increased likelihood of being affected [24]. Holmes et al. [23] demonstrated that many common orthopedic instruments produced noise levels greater than 85 dB (which is defined as a hazardous threshold). Similarly, Mullet et al. [25] and others have shown that many tools including saws, hammers, and drills have maximum levels up to 142 dB [24], [25], [26]. However, many of these studies have fallen short in that noise level has been evaluated in laboratory isolation and not in the context of performing surgery [27]. The current study addressed these shortcomings by performing noise evaluation during surgical intervention of the spine.

Noise generated by many surgical instruments used in spine surgery is considered impact noise, or noise generated by the collision of solid objects. Impact noise is considered much more harmful to hearing than continuous noise and these two have a synergistic effect when combined, placing exposed individuals at more significant risk of developing NIHL [28]. Exposure to these high-intensity impulses in and of themselves can cause acoustic trauma and instant mechanical damage to the ear [29]. Impact type noise is commonly measured by LCPeak on the C-weighted scale and is not to exceed 140dBC according to OSHA [30]. Peak level values approached this threshold in both cervical and thoracic/lumbar surgeries (121 dBC vs.120 dBC) with the maximum recorded LCPeak value of 132.9 dBC at the cusp of exceeding the damaging threshold. The synergistic effects of continuous operative noise with significant impact noise may place spine surgeons at elevated risk for the development of occupation related NIHL.

Kwan et al. recently demonstrated the increased risk of NIHL orthopedic surgeons endure [20,^21^,31]. Specifically, they demonstrated that spine surgeons are amongst those at highest risk for exposure to damaging levels of noise. Our study confirms these findings and showed an even higher risk of noise exposure. One hundred percent of spine surgeries evaluated had an MDL greater than 85 dBA, deemed to be a damaging level. Additionally, more than 3 quarters (78%) of all spine procedures had an MDL over 100 dBA. According to NIOSH recommendations, 100 dBA is a level of noise that is toxic if exposed to for a period greater than 15 minutes in 24 hours and the equivalent noise generated by a monster trucks [9,^32^,33]. The maximum MDL recorded was 111.5 dBA during a L5–S1 microdiscectomy. Although, further research is necessary to delineate portions of spine surgery which result in the highest risk for NIHL, the predilection for burr utilization during this case likely contributed to this significantly elevated noise level. Across the US, rates of spine surgery are increasing, with individual surgeon caseloads increasing to match [34], [35], [36], [37], [38], [39]. As such, spine surgeons are most often performing multiple surgeries per day. The cumulative noise risk from performing multiple surgeries a day, numerous times per week, with continued exposure to damaging levels of noise cannot be ignored.

This study demonstrates that spine surgeons are at significant risk of exceeding cumulative levels of sound with common operative exposures. Certain cases approach daily allowances within a singular surgical intervention. For example, the highest recorded dose of 72.9% means that the surgeon would exceed their allowed daily dose by 45.8% if they performed 2 of these cases in 1 day. Additionally, the highest projected dose was during a 3-level ACDF with duration of 52 minutes yielding a projected dose of 156%. The RELs would be exceeded if the surgeon operated with this level of sound exposure for just 5 hours.

The highest MDL was reached during 45-minutes microdiscectomy at 111.5 dBA, well above the 85 dB threshold where noise is considered damaging. This microdiscectomy had noise levels approaching those of a rock concert with irreversible hearing loss in less than 2 minutes of exposure [18]. Furthermore, 16% of cases had a projected dose greater than 50%. Performing two of these cases in a single (8-hour) operative day would cause the surgeon to exceed RELs. As spine surgeons often perform multiple procedures in a day, the risk of cumulative noise exposure exceeding daily RELs is significant and cannot be ignored.

This study is not without limitations. First, despite the microphone being worn either by the surgeon or resident staff in the front pocket, a sterile surgical gown is draped over the microphone itself (necessitated by sterile surgical protocol) which could cause a decrease in the magnitude of noise exposure. Additionally, it is possible that direct friction of the microphone against the surgical gown itself could contribute to noise caused by direct friction. However, this is likely minimal as the microphone was taped to users’ surgical lead/surgical scrubs to minimize motion. Second, with 5 spine surgeons participating in the current study this data may not display generalizability to a larger group of surgeons. This may be especially true as there exists a variety of ways to accomplish the same procedures with some surgeons favoring use of a burr whereas others preferring a Kerrison Rongeur for decompression. Also, the cases evaluated are some of the most commonly performed in spine surgery, but there are certain operative options which are under-represented in the current study. Third, in regards to the surgical suite, each OR has different sizes/configurations and acoustic characteristics which could affect sound generation. However, this is more likely applicable to other OR staff as compared with the surgeon who is consistently present directly next to the patient and surgical field. Fourth, most recordings taken had background music (94.1%) which was not at a controlled, constant volume. The contribution of background music to the intraoperative recordings is likely minimal as demonstrated by the “baseline with music” data shown in Table 2. However, it remains possible that the varied level of music in the operating suite could have contributed to noise exposure and cannot be ignored. Importantly, however, the microphone was consistently positioned closest to the surgical field and not the operating suite speakers. Fifth, the individual recording each surgical case was not consistent for all recordings thus height and distance from operative field was diverse. However, the microphone was consistently maintained within the operator's hearing zone which could make our findings more generalizable. Sixth, the NIOSH SLM application does not meet all international standards for professional sound measurement but rather serves as a practical tool to raise awareness about noise levels in the workplace [13]. For regulatory compliance, a formal noise survey conducted by an industrial hygienist or occupational and safety health specialist would be required. Seventh, given that the recording party maintained sterility throughout the procedure and the recording was continuous, there is no functionality to identify or correlate portions of the case which create the highest sound level and thus place staff at most risk for NIHL. Additionally, the NIOSH SLM app does not provide inflection point data with regards to specific instances of maximal sound exposure. Further investigation is warranted to delineate specific cases and precise portions of cases which place surgeons at highest risk for NIHL.

In conclusion, this study demonstrates that spine surgeons are regularly exposed to damaging levels of noise in the OR. Spine surgeons are routinely subject to noise levels over 100 dBA, far greater than the damaging threshold of 85 dBA. Although most cases individually did not exceed RELs, the cumulative effect of multiple operative cases every day with elevated noise cannot be ignored considering both impact and continuous noise. Noise protection for those present in the OR should be considered but needs to be evaluated in the context of communication. Noise protection cannot interfere with communication amongst operative staff as unhindered interaction is of critical importance in the surgical setting. Further research into a variety of spine procedures is necessary to better understand the true scope of the risk of NIHL. In the interim, it may be prudent for spine surgeons to consider daily case selection to limit noise exposure and the cumulative risk of NIHL.

## Declarations of Competing Interests

The authors declare that they have no known competing financial interests or personal relationships that could have appeared to influence the work reported in this paper.

## Appendix Supplementary materials

Image, application 1
